# 21. Efficacy and Safety of Maribavir as a Rescue Treatment for Investigator Assigned Therapy in Transplant Recipients With Refractory or Resistant Cytomegalovirus Infections in the SOLSTICE Study: Phase 3 Trial Results

**DOI:** 10.1093/ofid/ofab466.021

**Published:** 2021-12-04

**Authors:** Marcus Pereira, Carlos Cervera, Camille Kotton, Camille Kotton, Joseph Sasadeusz, Jingyang Wu, Martha Fournier

**Affiliations:** 1 Columbia University College of Physicians and Surgeons, New York, New York; 2 University of Alberta, Edmonton, AB, Canada; 3 Massachusetts General Hospital, Boston, MA; 4 Royal Melbourne Hospital, Melbourne, Victoria, Australia; 5 Shire Human Genetic Therapies, Inc., a Takeda company, Lexington, Massachusetts

## Abstract

**Background:**

Refractory or resistant (R/R) cytomegalovirus (CMV) infection after hematopoietic cell transplant (HCT) and solid organ transplant (SOT) cause serious, potentially fatal complications; therapeutic options are limited. In a Phase 3 study (NCT02931539), maribavir (MBV) was superior to investigator-assigned therapy (IAT; val/ganciclovir, foscarnet, cidofovir) for CMV clearance (Wk 8) and clearance plus symptom control (Wk 8 through Wk 16) in HCT/SOT recipients with R/R CMV infections. Here we present further study results on efficacy and safety of MBV in the rescue arm.

**Methods:**

Patients (pts) were stratified and randomized 2:1 to MBV (400 mg/bid) or IAT for 8-wk treatment then 12-wk follow-up. After minimum 3 wks’ treatment, pts in the IAT group meeting criteria (worsening/lack of improvement of CMV infection or failure to achieve viremia clearance plus IAT intolerance) could enter a MBV rescue arm (8-wk treatment, 12-wk follow-up). In the rescue arm, efficacy was evaluated by confirmed CMV viremia clearance (plasma CMV DNA < 137 IU/mL in 2 consecutive tests ≥ 5 days apart) at end of Wk 8 and confirmed clearance with symptom control at Wk 8 through Wk 16. Safety was assessed.

**Results:**

A total of 352 pts were randomized (MBV: 235, IAT: 117, randomized set). Confirmed CMV viremia clearance at Wk 8 was achieved in 131 (55.7%) and 28 (23.9%) pts, respectively, in the randomized set. Having met criteria, 22 (18.8%) pts entered the MBV rescue arm; at entry, 6 (27.3%) pts had developed neutropenia and 9 (40.9%) had increased serum creatinine (**Table 1**). At Wk 8 of rescue therapy, 11 (50.0%) pts achieved confirmed CMV viremia clearance; 6 (27.3%) pts had CMV clearance with symptom control at Wk 8 maintained through Wk 16 (**Table 2**). All 22 pts reported treatment-emergent adverse events (TEAEs; **Table 3**); most common TEAEs of special interest were nausea, vomiting, and diarrhea (54.5%), and taste disturbance (50.0%). Neutropenia and acute kidney injury TEAEs were reported by 0 and 3 pts in the rescue arm, respectively.

Table 1. Summary of patients from IAT-randomized group meeting criteria for entry into MBV rescue arm*

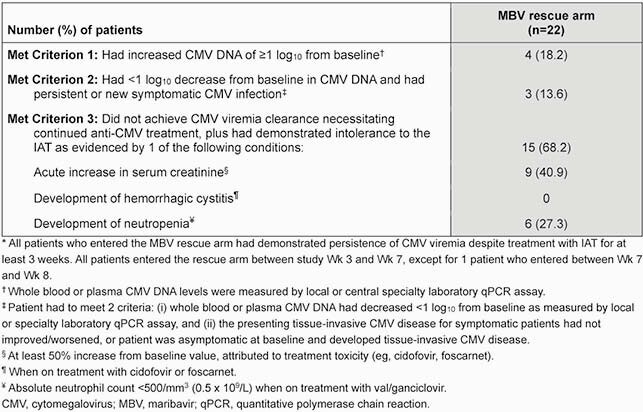

Table 2. Patients achieving confirmed CMV viremia clearance at end of Wk 8 (end of treatment) or achieving confirmed CMV viremia clearance and symptom control at end of Wk 8 maintained through Wk 16

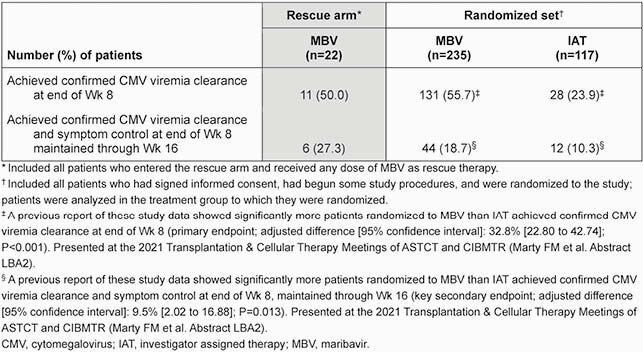

Table 3. Treatment-emergent adverse events during the on-rescue observation period

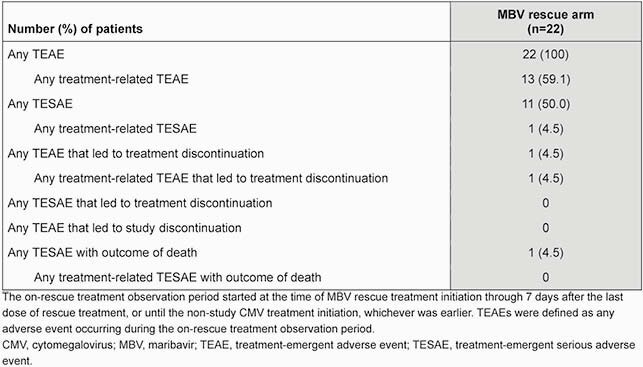

**Conclusion:**

Rescue arm data show MBV was efficacious for R/R CMV infection in HCT/SOT recipients inadequately responding to IAT with/without intolerance and had a similar safety profile to that reported for pts in the randomized MBV group.

**Disclosures:**

**Marcus Pereira, MD**, **Hologic** (Scientific Research Study Investigator)**Merck** (Scientific Research Study Investigator)**Takeda** (Scientific Research Study Investigator, Advisor or Review Panel member) **Carlos Cervera, MD, PhD**, **Avir Pharma** (Consultant, Advisor or Review Panel member)**Lilly** (Consultant, Advisor or Review Panel member)**Merck** (Consultant, Advisor or Review Panel member, Research Grant or Support)**Sunovion** (Consultant, Advisor or Review Panel member)**Takeda** (Consultant, Advisor or Review Panel member)**Veritas Pharma** (Consultant, Advisor or Review Panel member) **Camille Kotton, MD**, **Shire/Takeda** (Advisor or Review Panel member) **Camille Kotton, MD**, UpToDate (Individual(s) Involved: Self): I write chapters on zoonoses for UpToDate., Independent Contractor **Joseph Sasadeusz, MBBS, PhD**, **Abbvie** (Grant/Research Support, Other Financial or Material Support, Consulting fee: speaker)**Gilead** (Other Financial or Material Support, Speaker)**Merck** (Grant/Research Support, Consulting fee: speaker)**Takeda** (Grant/Research Support) **Jingyang Wu, MS**, **Shire Human Genetic Therapies, Inc., a Takeda company** (Employee, Other Financial or Material Support, Holds stock/stock options) **Martha Fournier, MD**, **Shire Human Genetic Therapies, Inc., a Takeda company** (Employee, Other Financial or Material Support, Holds stock/stock options)**Shire ViroPharma, a Takeda company** (Other Financial or Material Support, This study was funded by Shire ViroPharma, a Takeda company)

